# Application of dermoscopy for efficacy assessment and prognosis in laser treatment of vasodilator dermatosis

**DOI:** 10.3389/fmed.2025.1703471

**Published:** 2025-12-16

**Authors:** Xiaoye Liu, Manli Qi, Huijuan Wang, Yajun Zhang, Guoqiang Zhang, Weiwei Xu

**Affiliations:** 1 The First Hospital of Hebei Medical University, Shijiazhuang, China; 2 Department of Dermatology, Tianjin Union Medical Center, The First Affiliated Hospital of Nankai University, Tianjin, China

**Keywords:** long-pulsed 1064 nm laser, dermoscopy, vascular dermatoses, treatment efficacy, predictive factors

## Abstract

**Objective:**

Analyse dermoscopic image data before and after laser treatment for vasodilator dermatosis. Investigate its value in evaluating post-treatment efficacy and identifying factors predictive of complete lesion clearance in a single session.

**Methods:**

Patients undergoing laser treatment for vascular skin disorders in our department were selected. Dermoscopic images of the lesions were captured and the vascular characteristics within the dermoscopic field were recorded. One-month follow-up analyses assessed lesion clearance status and dermoscopic features in each patient.

**Results:**

This study included 18 patients (37 lesions in total). The total vascular feature scores before and after treatment were significantly different from the individual vascular scores. The single-session clearance rate at 1-month follow-up was 75.7% (28/37). Significant differences in scores were observed between the cleared and uncleared groups. Univariate and multivariate logistic analyses identified vascular color as an independent factor influencing lesion clearance. Calculating the Youden index showed that lesions with scores below 9.5 were more likely to be cleared in a single session.

**Conclusion:**

Prior to laser treatment, using a dermatoscope to comprehensively assess the characteristics of vascular structures provides an objective evaluation of the underlying vascular condition. This makes it possible to predict the likelihood of achieving complete clearance in a single session after laser therapy, providing insights into how to devise personalized treatment plans for patients. The clinical promotion and application of this method is eagerly awaited.

## 1 Introduction

Vasodilator dermatosis refers to conditions that arise from the persistent dilation of capillaries or micro-arteriovenous vessels in the skin or mucous membranes. These conditions are clinically manifested as red to purple macules, plaques, linear lesions or spider angiomas (1). Previous treatments have included topical or oral medications, local sclerotherapy injections and physical methods such as surgery, electrocautery, microwave therapy and cryotherapy (2–4). However, with advancements in laser technology and equipment, non-invasive laser therapy has emerged as the preferred treatment worldwide due to its favorable safety profile and efficacy (1).

Currently, there is a lack of objective clinical assessment criteria for evaluating the efficacy of treatments for telangiectatic dermatoses. Traditional methods rely heavily on physicians’ subjective visual comparisons of pre- and post-treatment photographs, which are susceptible to variations in lighting, environmental conditions, and physician experience (5). Although pathological examination is considered the “gold standard,” it is limited in its application for laser efficacy assessment due to its invasive nature, poor reproducibility and inability to provide dynamic observation. Consequently, the urgent priority is to establish a safe, non-invasive imaging assessment system that can objectively evaluate and even predict laser treatment outcomes. Dermoscopy is a non-invasive diagnostic tool that clearly visualizes minute skin structures to aid the diagnosis of vascular disorders. By quantitatively analyzing parameters such as vascular morphology, mean diameter and color, and conveniently documenting pre- and post-treatment changes, it has the potential to provide a non-invasive assessment (6). This study introduces dermoscopy technology for imaging and follow-up before and after laser treatment for vascular dilation skin diseases. It aims to explore objective factors influencing efficacy, preliminarily predict the likelihood of single-session clearance, and provide objective evidence for doctor-patient communication.

## 2 Materials and methods

### 2.1 Clinical data

Patients with vascular dilation skin diseases who were treated with long-pulsed 1064 nm laser therapy at the Tianjin Medical University General Hospital between March 2023 and March 2024 were enrolled in this study according to the specified inclusion and exclusion criteria. This study was approved by the Tianjin Medical University General Hospital’s Medical Ethics Committee. Written informed consent for participation was obtained from the participants or the participants’ legal guardians/next of kin.

#### 2.1.1 Inclusion criteria

(1)    Patients who were clinically diagnosed with simple telangiectasia, haemangioma or spider angioma, with no restrictions on age or gender.
(2)    Patients able to undergo laser treatment, with no restrictions on lesion location or type.
(3)    Patients in generally good health with no history of systemic diseases.
(4)    No prior treatment for the skin lesions.

#### 2.1.2 Exclusion criteria

1. Patients with other skin conditions and/or lesions at the affected site.
2. Patients with a keloid-prone constitution, epilepsy, or who are taking photosensitizing medications or have photosensitive dermatoses are excluded.

### 2.2 Treatment and scoring methods

#### 2.2.1 Dermoscopy examination

The same clinician performed dermoscopic examinations using a standardized protocol under consistent lighting conditions. Images were captured using a polarized light (PL) dermoscope (BN-TBWG-1,001, Nanjing Beijing Medical Equipment Co., Ltd.). These pictures showed vascular density, color, width and vascular pattern. Immediately following laser treatment, a repeat dermoscopy examination of the same area was conducted to record vascular characteristics.

#### 2.2.2 Laser treatment

The clinician employed the long-pulsed 1,064 nm vascular laser handpiece from the Israeli Fotona Brilliant workstation and selected treatment parameters based on lesion characteristics. The treatment endpoint was defined as either blurred, dilated vessels or a shift in color from red to grayish-white within the treatment area, provided that there was no vascular rupture. After treatment, ice packs were applied for 15–20 min to reduce redness, swelling and discomfort.

#### 2.2.3 Efficacy assessment

Two additional physicians independently documented the pre- and post-treatment dermoscopic features of vascular density, diameter, color and pattern (7). Quantitative statistics were performed, with individual vascular parameters scored and totaled to yield the lesion’s overall dermoscopic score.

##### 2.2.3.1 Vascular density

**FIGURE 1:**
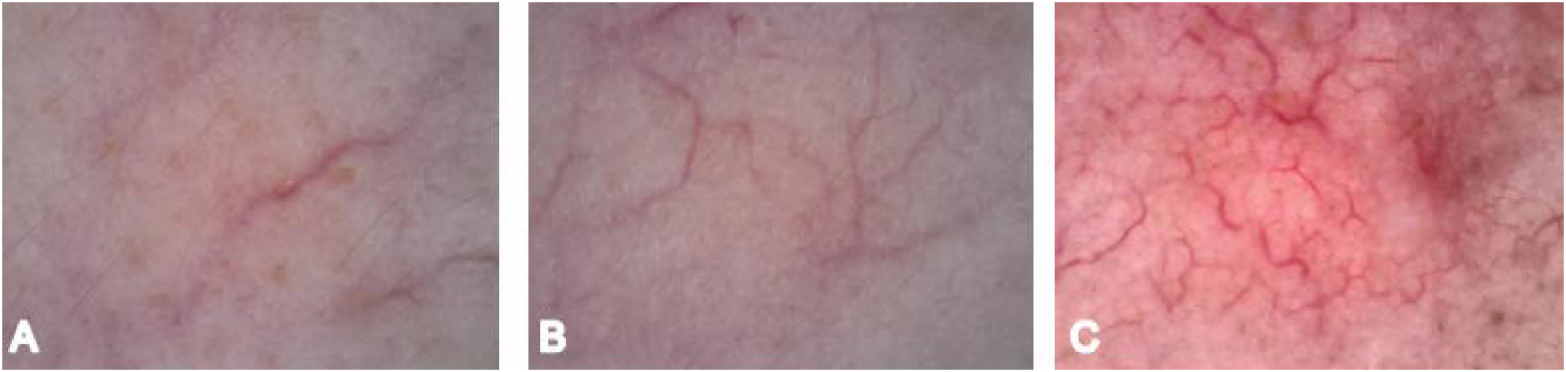
Vascular density scoring. **(A)** Vascular density 1. **(B)** Vascular density 2. **(C)** Vascular density 3.

Distribution definitions: 1 = sparse (fewer than 5 vessels in the entire field of view), 2 = moderate (5–10 vessels in the entire field of view), 3 = dense (more than 10 vessels in the entire field of view) (7) (Figure 1).

##### 2.2.3.2 Vascular diameter

**FIGURE 2:**
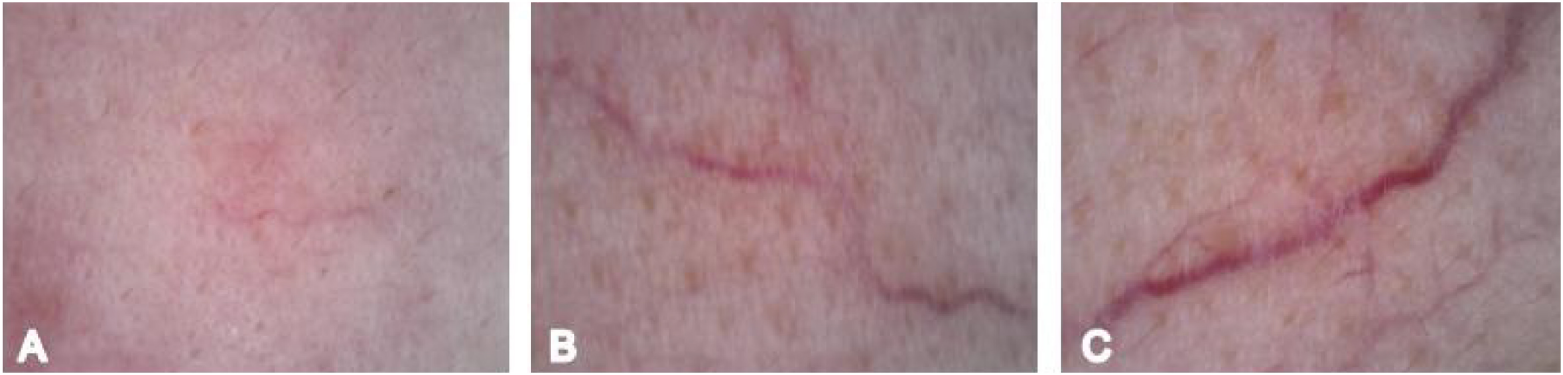
Vascular diameter scoring. **(A)** Vessel diameter 1. **(B)** Vessel diameter 2. **(C)** Vessel diameter 3.

Vascular diameter distribution is defined as follows: 1 = Small vessels (dermoscopic diameter ≤ 2 mm), 2 = Medium vessels (2 mm < diameter < 4 mm), 3 = Large vessels (diameter ≥ 4 mm) (7) (Figure 2).

##### 2.2.3.3 Vascular color

**FIGURE 3:**
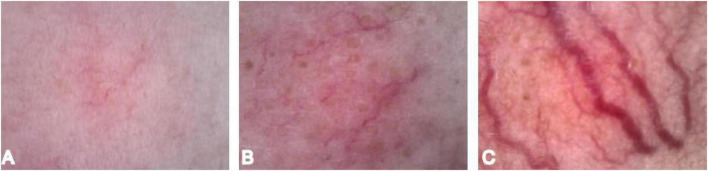
Vascular color scoring. **(A)** Vessel color 1. **(B)** Vessel color 2. **(C)** Vessel color 3.

Vascular color is categorized by intensity as 1 = pink, 2 = red, 3 = purple (7) (Figure 3).

##### 2.2.3.4 Vascular pattern

**FIGURE 4:**
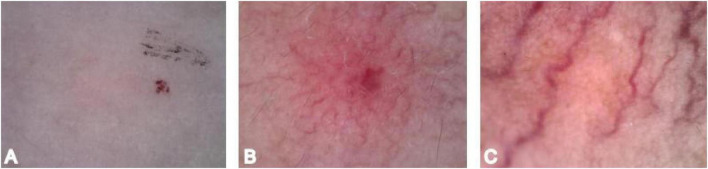
Vascular pattern scoring. **(A)** Vascular pattern 1. **(B)** Vascular pattern 2. **(C)** Vascular pattern 3.

1 = Superficial vascular pattern comprising punctate, globular, or sausage-shaped vessels; 2 = Mixed vascular pattern where both superficial and deep vascular patterns are observable; 3 = Deep vascular pattern comprising linear, branched, or dendritic vessels (8) (Figure 4).

### 2.3 Statistical analysis

A *t*-test was used to analyze changes in dermoscopic lesion scores before and after laser treatment. Univariate and multivariate logistic regression analyses were performed to determine whether vascular characteristics constituted independent factors influencing single-session clearance rates. The area under the ROC curve was then used to identify the dermoscopic vascular features that were most significant in affecting single-session clearance rates post-treatment.

## 3 Results

### 3.1 General data

This study included 18 patients (37 lesions in total). Seven patients were male (15 lesions) and 11 were female (22 lesions). The lesions comprised 25 cases of telangiectasia, seven cases of haemangioma and five cases of spider angioma.

### 3.2 Treatment efficacy before and after lesion management

The results are presented in Tables 1, 2. The mean total vascular feature score for all 37 lesions was 8.65 ( ± 1.549) before treatment and 7.11 ( ± 1.242) after treatment. A *t*-test revealed statistically significant differences (*p* < 0.001). Individual vascular feature scores also showed statistically significant differences when analyzed using a paired-samples rank-sum test (*p* < 0.001).

**TABLE 1:** Differences in total vascular feature scores across 37 lesions before and after treatment.

Indicator	Number of lesions	Pre-treatment	After treatment	Difference	*t*	*p*
Total score	37	8.65 ± 1.55	7.11 ± 1.24	1.54 ± 0.39	8.017	<0.001

**TABLE 2:** Differences in vascular characteristic scores at 37 skin lesion sites before and after treatment.

Indicator	Number of lesions	Before treatment	After treatment	Difference	*Z*	*P*
Vascular density	37	1.76 ± 0.83	1.32 ± 0.58	0.43 ± 0.69	−3.176	*P* = 0.001
Vessel diameter	37	2.11 ± 0.88	1.68 ± 0.75	0.43 ± 0.56	−3.771	*P*<0.001
Vascular color	37	2.30 ± 0.62	1.62 ± 0.68	0.68 ± 0.58	−4.630	*P*<0.001

### 3.3 Single-session clearance rate of skin lesions

Following a single laser treatment for 37 cases of telangiectatic dermatosis, the 1-month follow-up revealed a single-session clearance rate of 75.7%, with 28 cases showing complete clearance and 9 cases showing incomplete clearance.

### 3.4 Distribution of dermatoscopic scores prior to lesion treatment

Results are presented in Table 3. After 1 month, the mean total dermoscopic score for the 28 cleared lesions was 8.10 ( ± 1.113), whereas the nine uncleared lesions scored 10.75 ( ± 0.886). This difference was statistically significant (*P* < 0.001).

**TABLE 3:** Differences in total vascular score between cleared and uncleared lesions after 1 month.

Indicator	Number of lesions	Pre-treatment	*F*	*Z*	*P*
Clearance	28	8.10 ± 1.11	0.14	−6.679	*P*<0.001
Not cleared	9	10.75 ± 0.89			

### 3.5 Logistic regression analysis of dermoscopic scores for lesions cleared and not cleared in a single session

The results are presented in Tables 4, 5. One month after treatment, univariate logistic regression analysis showed that the vascular diameter and color of lesions as seen under dermoscopy were statistically significant factors affecting single-session clearance (*p* < 0.05). Further analysis using multivariate logistic regression identified vascular color as an independent predictor of lesion clearance (P < 0.05).

**TABLE 4:** Univariate logistic regression analysis of dermoscopic vascular characteristics between cleared and uncleared groups.

Indicator	β	S.E.	Wald χ^2^value	df	*p*	OR	95% CI	
							Lower bound	Upper limit
Vascular density	0.919	0.490	3.512	1	0.061	2.506	0.959	6.553
Vessel diameter	1.221	0.599	4.154	1	0.042	3.390	1.048	10.965
Vascular color	2.245	0.892	6.332	1	0.012	9.438	1.643	54.224
Vascular pattern	19.010	8,007.994	0.000	1	0.998	180,359,098.249	0.000	

**TABLE 5:** Multivariate logistic regression analysis of dermoscopic vascular characteristics between the clearance group and non-clearance group.

Indicator	β	S.E.	Wald χ^2^value	df	*p*	OR	95% CI	
							Lower bound	Upper limit
Vessel diameter	−1.011	0.712	2.016	1	0.156	0.364	0.090	1.469
Vascular color	−2.001	0.938	4.547	1	0.033	0.135	0.021	0.851

### 3.6 Area under the ROC curve statistics

**FIGURE 5:**
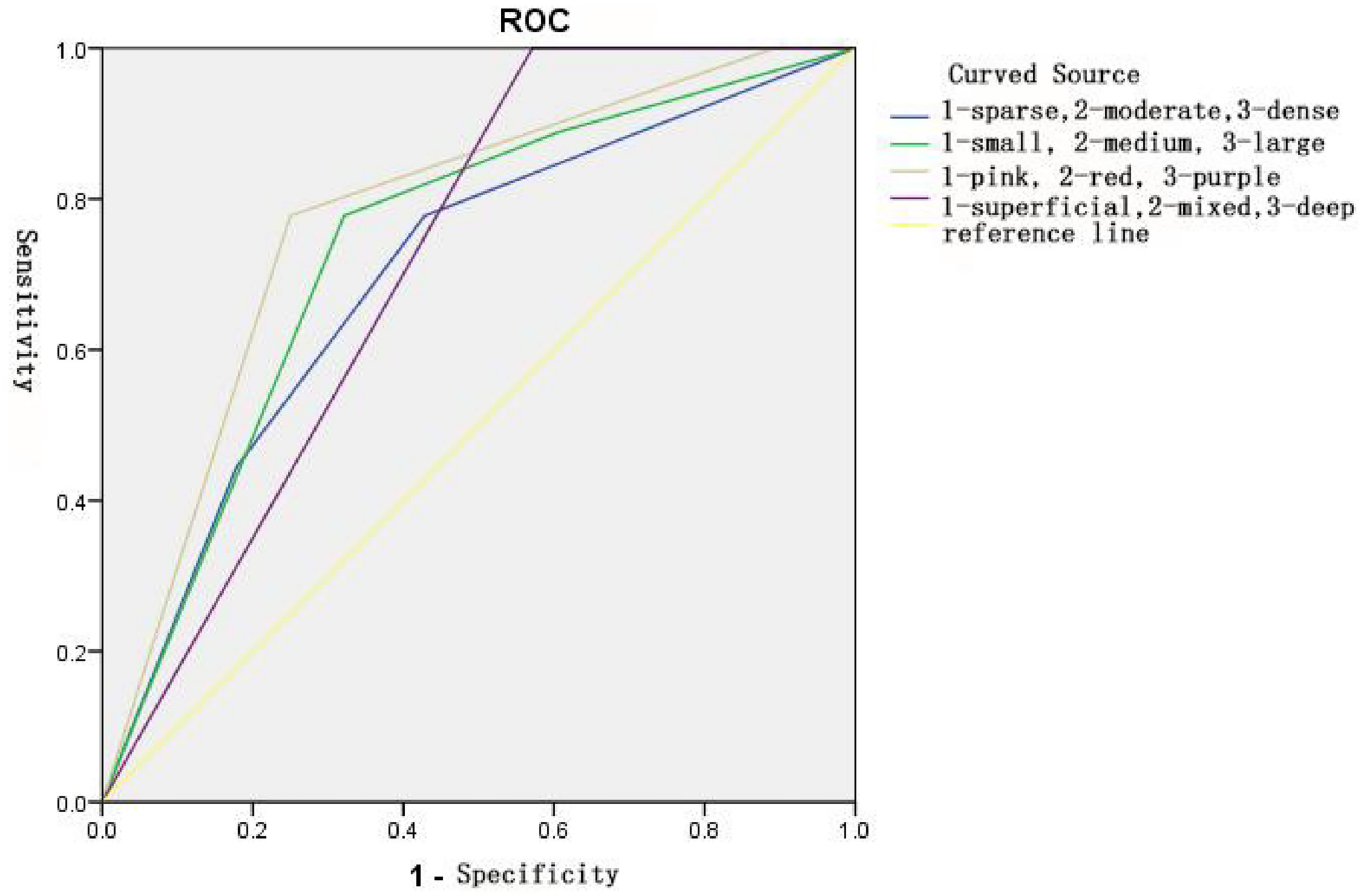
Area under the ROC curve for dermoscopic vascular feature scores between the clearance and non-clearance groups.

**FIGURE 6:**
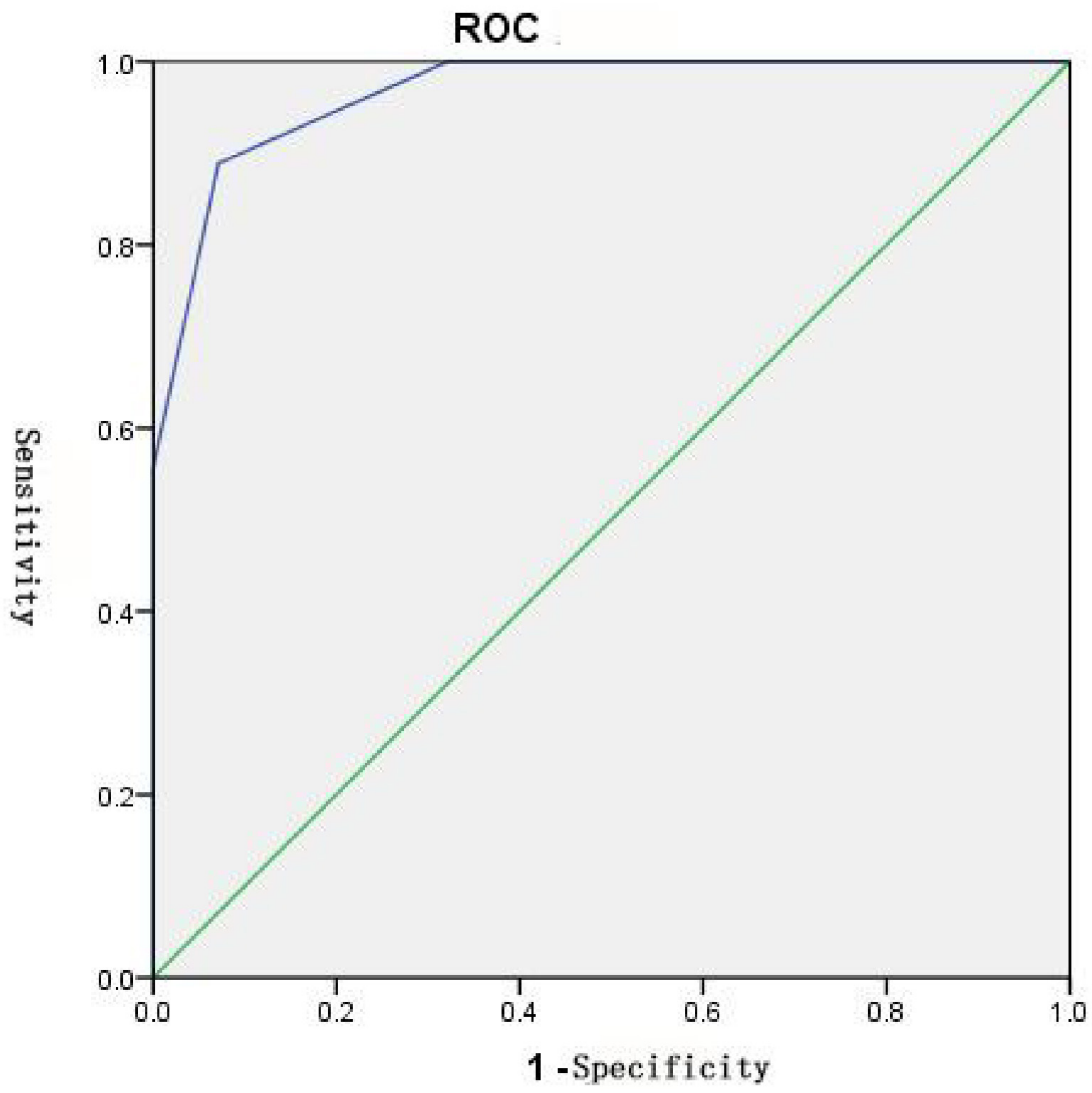
Statistical graph of the area under the ROC curve for the total dermoscopic vascular score between the cleared and uncleared groups.

The results are presented in Figures 5, 6 and Tables 6, 7. One month after laser treatment, comparisons between cleared and non-cleared lesions revealed ROC curve areas exceeding 0.5 for all dermoscopic vascular features. There were statistically significant differences in vascular color and diameter (*P* < 0.05). The ROC curve area for the total vascular score also exceeded 0.5, with statistically significant differences (*P* < 0.05). When the maximum Yorden index was calculated, the vascular color score was 2.5, the vascular diameter score was 2.5 and the total vascular feature score was 9.5. These results suggest that lesions are more easily cleared in a single session when the vascular color and diameter scores are both below 2.5 or the total vascular feature score is below 9.5.

**TABLE 6:** Statistical analysis of the area under the ROC curve for dermoscopic vascular feature scores between the cleared and uncleared groups.

Indicator	Area	S.E.	*p*	95% CI	
				Lower bound	Upper limit
Vascular density	0.700	0.101	0.074	0.502	0.899
Vessel diameter	0.734	0.095	0.037	0.549	0.920
Vascular colour	0.776	0.088	0.014	0.603	0.948
Vascular pattern	0.714	0.085	0.056	0.548	0.880

**TABLE 7:** Area under the ROC curve for total dermoscopic vascular score between cleared and uncleared groups.

Indicator	Area	S.E.	*p*	95% CI	
				Lower bound	Upper limit
Total score	0.966	0.028	0.000	0.911	1.000

## 4 Discussion

Vasodilator dermatosis are characterized by red to purple vascular globules or linear dilations. When they occur on areas of the body that are exposed, such as the face, they often cause distress and cosmetic concerns for patients, necessitating active treatment. Laser therapy is currently the primary treatment approach for telangiectatic skin conditions, both domestically and internationally. The energy it generates induces the degeneration and necrosis of vascular endothelial cells without damaging the surrounding healthy tissue. Lasers operating at 1,064 nm have a greater penetration depth and pulse width, which yields favorable outcomes for deeper lesions with larger vessel diameters. Khamaysi et al. (9) treated 30 propranolol-unresponsive haemangioma patients using 1,064 nm laser therapy, achieving varying degrees of improvement. Literature (10) demonstrated that 78.6% of 145 patients exhibited significant ( > 75%) improvement following 1,064 nm laser therapy, with 80% patient satisfaction. Adverse reactions such as blistering and scarring following laser treatment are primarily caused by the incorrect selection of energy levels. Therefore, it is crucial to thoroughly understand the vascular characteristics prior to laser therapy in order to adjust parameters and predict efficacy, thereby effectively avoiding unnecessary disputes.

**FIGURE 7:**
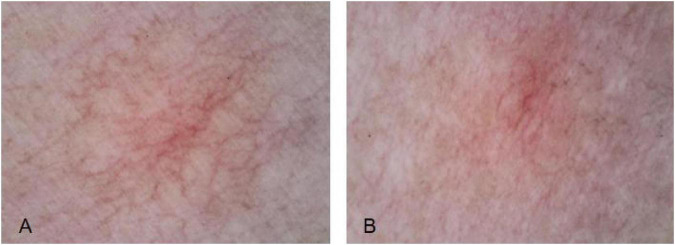
Vascular density before and after LP1064 nm laser treatment. **(A)** Before treatment. **(B)** After treatment.

**FIGURE 8:**
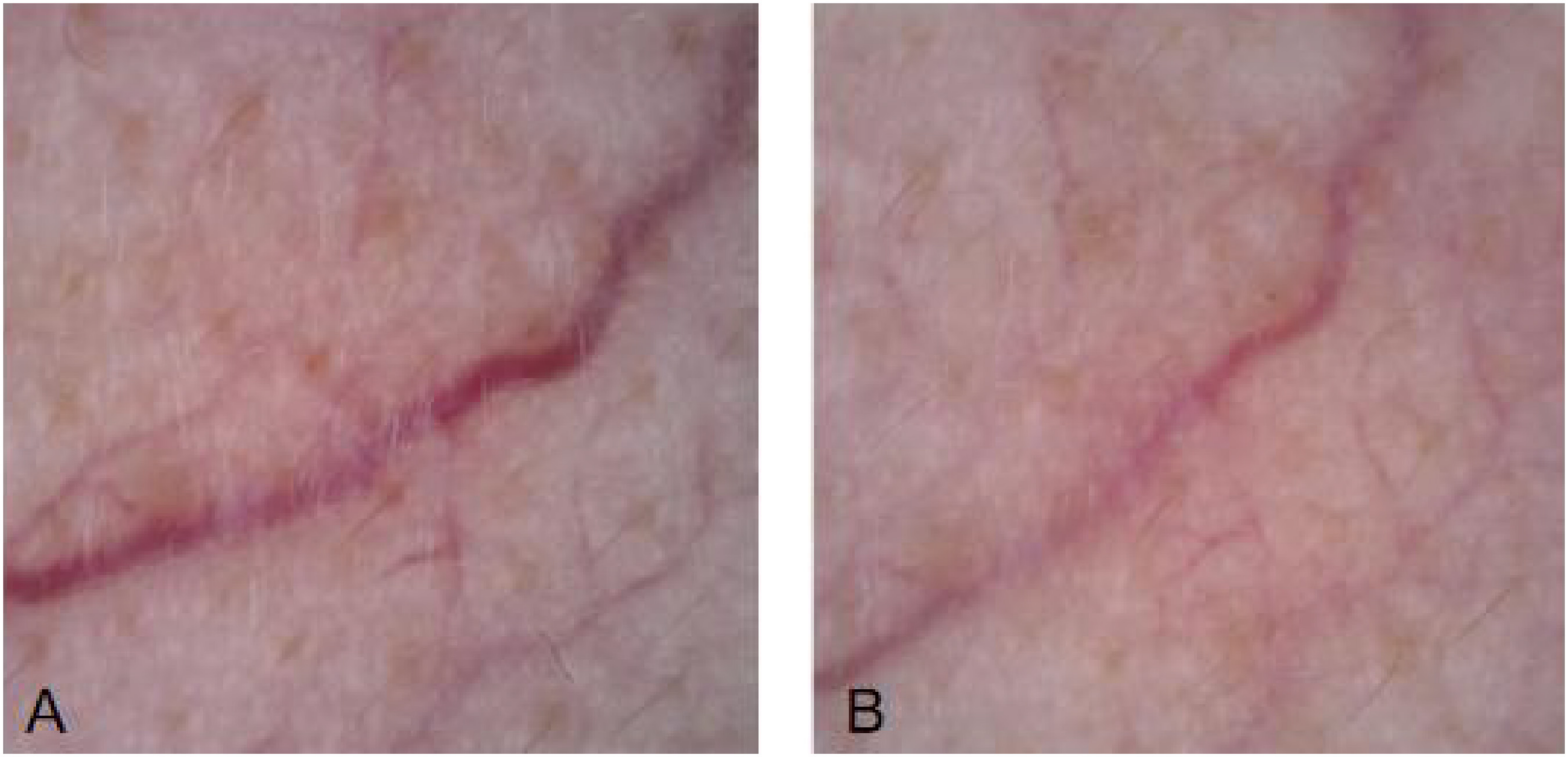
Vascular diameter before and after LP1064 nm laser treatment. **(A)** Before treatment. **(B)** After treatment.

**FIGURE 9:**
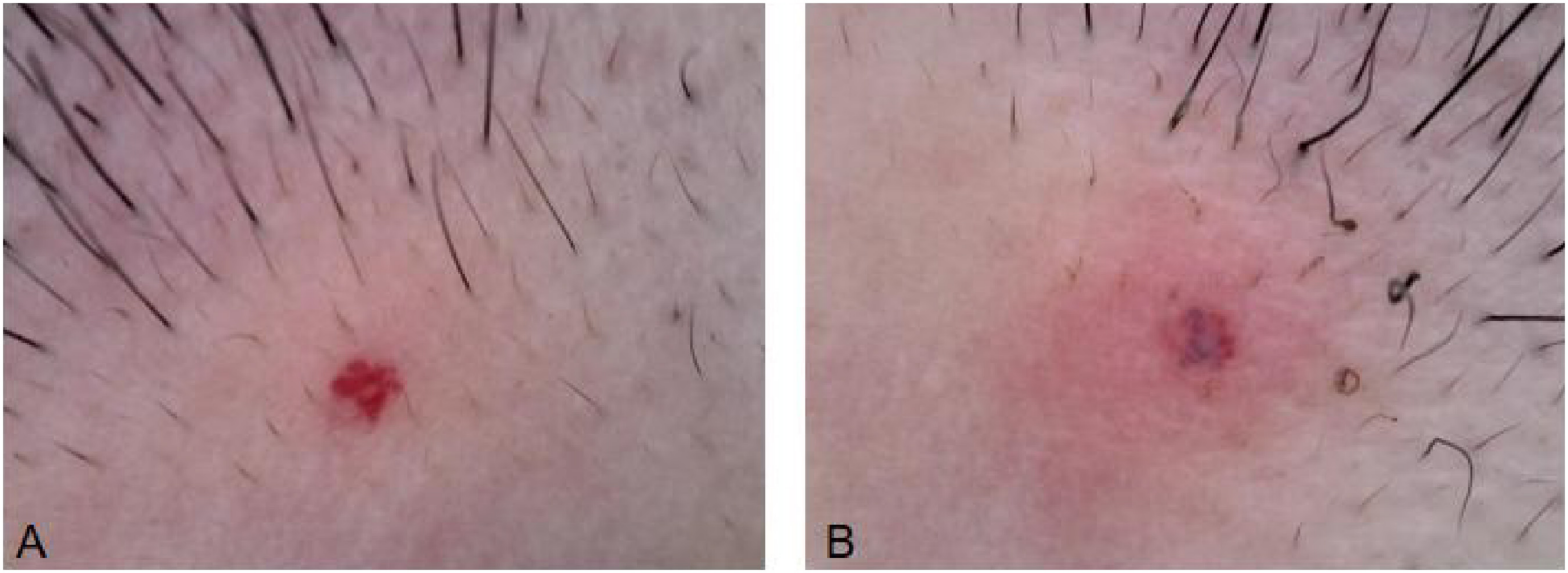
Vascular color before and after LP1064 nm laser treatment. **(A)** Before treatment. **(B)** After treatment.

This study compared the dermatoscopic appearance of telangiectatic skin conditions before and immediately after laser treatment. It concluded that vascular density, vessel diameter and vascular color all improved to varying degrees after treatment. This further validates the efficacy of laser therapy for these conditions, demonstrating its immediate vasoconstrictive effect. Clinicians can also use pre- and post-treatment dermatoscopic lesion morphology to make a preliminary assessment of treatment outcomes (Figures 7–9).

This study analyzed 18 patients (37 lesions), achieving a clearance rate of 75.7% (28/37) in a single session. Lesions that were not completely cleared predominantly exhibited dense vascularity, a diameter of more than 4 mm, a purplish-red color, and an exclusively linear vascular pattern. Pre-treatment dermatoscopic scoring enabled the preliminary prediction of treatment efficacy. Univariate logistic analysis revealed that vessel diameter and color significantly affected single-session clearance. Multivariate logistic analysis revealed that vessel color is an independent predictor of clearance, which is potentially related to oxyhaemoglobin content. Previous studies have suggested that higher oxyhaemoglobin levels correlate with a more vivid red color and relatively smaller vessel diameters (11).

ROC curve analysis showed that the AUC for all vascular feature indicators was greater than 0.5, in the order of color > diameter > density > pattern. Color and diameter demonstrated significant predictive value. The AUC for the total vascular feature score was 0.966, indicating that the cumulative vascular feature score is crucial for predicting single-session lesion clearance. A Youden index of 9.5 was determined; scores below this threshold suggest a high likelihood of single-session clearance, whereas higher scores indicate the need for multiple treatments. Pre-treatment observation of vascular diameter and color under dermatoscopy can guide the subsequent selection of treatment parameters to a certain extent.

This study introduces an innovative application of dermoscopy for the quantitative assessment of vascular characteristics, prediction of treatment efficacy, and reduction of subjective bias. However, the visibility of deep vessels is limited by dermatoscopy; future research may therefore integrate non-invasive imaging techniques, such as skin CT or ultrasound.

This study has several limitations, including a relatively small sample size and a short follow-up period. Future research will involve expanding the sample size, extending the follow-up duration, and monitoring clearance, recurrence, and adverse events to enable a more comprehensive analysis of the data.

In summary, using dermatoscopy to comprehensively assess the characteristics of vascular structures before treating vasodilator dermatosis with long-pulsed 1,064 nm laser therapy provides an objective and accurate understanding of the characteristics of the vascular structures to be treated and enables prediction of the likelihood of achieving complete clearance in a single session. This further validates the feasibility of using dermatoscopy to assess the therapeutic efficacy of, and predict the outcomes of, long-pulsed 1,064 nm vascular laser therapy for telangiectatic skin conditions. It preliminarily demonstrates its superiority in the quantitative evaluation of efficacy, offering insights and methodologies for the development of personalized treatment plans for future patients. Further clinical validation and application are warranted.

## Data Availability

The raw data supporting the conclusions of this article will be made available by the authors, without undue reservation.
